# Risk Estimation with Epidemiologic Data When Response Attenuates at High-Exposure Levels

**DOI:** 10.1289/ehp.1002521

**Published:** 2011-01-10

**Authors:** Kyle Steenland, Ryan Seals, Mitch Klein, Jennifer Jinot, Henry D. Kahn

**Affiliations:** 1 Rollins School of Public Health, Emory University, Atlanta, Georgia, USA; 2 National Center for Environmental Assessment, U.S. Environmental Protection Agency, Washington, District of Columbia, USA

**Keywords:** ethylene oxide, risk assessment, statistical models

## Abstract

**Background:**

In occupational studies, which are commonly used for risk assessment for environmental settings, estimated exposure–response relationships often attenuate at high exposures. Relative risk (RR) models with transformed (e.g., log- or square root–transformed) exposures can provide a good fit to such data, but resulting exposure–response curves that are supralinear in the low-dose region may overestimate low-dose risks. Conversely, a model of untransformed (linear) exposure may underestimate risks attributable to exposures in the low-dose region.

**Methods:**

We examined several models, seeking simple parametric models that fit attenuating exposure–response data well. We have illustrated the use of both log-linear and linear RR models using cohort study data on breast cancer and exposure to ethylene oxide.

**Results:**

Linear RR models fit the data better than do corresponding log-linear models. Among linear RR models, linear (untransformed), log-transformed, square root–transformed, linear-exponential, and two-piece linear exposure models all fit the data reasonably well. However, the slopes of the predicted exposure–response relations were very different in the low-exposure range, which resulted in different estimates of the exposure concentration associated with a 1% lifetime excess risk (0.0400, 0.00005, 0.0016, 0.0113, and 0.0100 ppm, respectively). The linear (in exposure) model underestimated the categorical exposure–response in the low-dose region, whereas log-transformed and square root–transformed exposure models overestimated it.

**Conclusion:**

Although a number of models may fit attenuating data well, models that assume linear or nearly linear exposure–response relations in the low-dose region of interest may be preferred by risk assessors, because they do not depend on the choice of a point of departure for linear low-dose extrapolation and are relatively easy to interpret.

A key goal of risk assessment is to develop a quantitative exposure–response model from which excess risk of disease can be estimated for any given exposure. Increasingly, human epidemiologic data are being used to construct these models that form the basis for risk estimates. When appropriate epidemiologic studies with good exposure–response data are available, these data are usually preferred over laboratory animal data for risk assessment, because animal data introduce additional uncertainty due to cross-species extrapolation. Here we discuss risk assessment methods using epidemiologic data with a dichotomous disease outcome and observations across a variety of exposure levels, such that exposure–response modeling is possible, with cumulative exposure as the exposure metric of interest (as is common for chronic disease). We use a variety of relative risk (RR) models, both log-linear (log RR = β_1_ × exposure; no intercept term β_0_ is fit in Cox regression) and linear (of the form RR = 1+ β × exposure). The models can include covariates and different transformations of functions of exposure. Linear RR models (sometimes called excess RR models) have some advantages, that is, they are more easily interpretable than are log RR models, and they provide a straight line to the origin in the low-dose region.

When using human cancer data, the U.S. Environmental Protection Agency (EPA) often calculates the estimated lifetime exposure concentration that results in an excess risk of disease of 1% [exposure concentration (EC_01_)], although other smaller ECs are sometimes used (U.S. EPA 2005). Larger ECs (e.g., EC_05_ or EC_10_) are generally used for rodent studies of carcinogens; however, for human cancer studies, extra risk seldom rises to such levels in the observable range of the data, which is especially true for rare cancers, such as nasopharyngeal cancer in relation to formaldehyde. The U.S. EPA then uses the exposure concentration corresponding to the 95% lower bound on the EC_01_ (i.e., the LEC_01_) as a point of departure for low-dose (low-exposure) linear extrapolation to estimate the risk associated with a one-unit increase in exposure (or dose). The LEC_01_ is essentially the same as the benchmark dose lower limit. Linear low-dose extrapolation from the point of departure to the origin is generally used to estimate low-dose risks for carcinogens, unless a nonlinear mode of action has been established (U.S. EPA 2005). The U.S. EPA uses the following formula to calculate excess risk (or extra risk) for a given level of exposure:


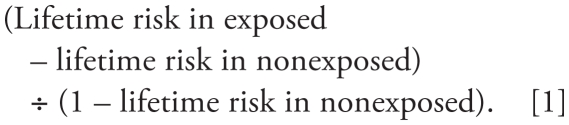


The background risk of the disease of interest (i.e., the lifetime risk in the nonexposed) is subtracted from 1 in the denominator to scale the excess risk to the population expected to be free of background disease, a procedure used conventionally with rodent data.

Lifetime risks for the exposed and nonexposed populations are calculated using actuarial methods in which mortality from other causes is taken into account. Exposure is assumed to be constant over time, and hence cumulative exposure increases over time. Age-specific background rates for disease in the nonexposed are multiplied by rate ratios estimated from the epidemiologic exposure–response model for given cumulative exposure levels to get age-specific rates of disease in the exposed, and lifetime risks for both exposed and nonexposed are calculated across ages.

Under certain circumstances, estimates of the exposure concentration corresponding to a 1% excess risk can be highly dependent on the exposure–response model chosen to model the epidemiologic data. One such case where this occurs is when the observed exposure–response data level off or plateau (attenuate) at higher exposures, as has been observed frequently in occupational epidemiology. For example, this phenomenon has been seen for cancer in relation to dioxin, silica, 1,3-butadiene, cadmium, beryllium, radon daughters, diesel fumes, nickel, arsenic, ethylene oxide (EtO), and hexavalent chromium (Stayner et al. 2003). Such attenuation also has been observed in some studies of noncancer outcomes, for example, silica and kidney disease, and silica and silicosis mortality (e.g., Steenland et al. 2001; ’t Mannetje et al. 2002).

A number of reasons for such an attenuation effect have been advanced, including depletion of susceptible individuals in the population, saturation of biological processes, misclassification of exposure at high levels, and the healthy worker–survivor effect (Stayner et al. 2003). Typically, models that provide a good fit to the data in this situation, such as models in which the exposure is log transformed or square root transformed (e.g., power models), are supralinear in the low-exposure region such that slope of the exposure–response curve is high at low exposures (see Rothman et al. 2008 for a discussion of transformation of exposure variables). This high slope in the low-exposure region of interest results in EC_01_s that are very low. Furthermore, without linearity or near linearity in the low-dose region, the slope of the linear low-exposure extrapolation used for risk assessment is very sensitive to the particular point of departure (e.g., LEC_01_ vs. LEC_001_) used for low-dose extrapolation. On the other hand, a model with a simple untransformed linear term for cumulative exposure may not fit the attenuating data as well and may underestimate risk in the low-dose region, resulting in excessively large estimates of EC_01_.

One alternative is to use a two-piece spline model, that is, a spline curve with one knot. Splines are piecewise polynomials of degree *n*, the simplest being a linear spline of degree 1. The point at which the pieces join is called a knot. Splines can be especially useful in approximating the shapes of exposure–response curves, which may vary substantially over the range of exposure (see Rothman et al. 2008 for a discussion of the application of splines in the analysis of epidemiological data). In our approach, the data are used to fit log-linear or linear-risk models with two linear sections or pieces, which permit the exposure–response relation to have a higher slope in the lower exposure region than in the higher exposure region when it is attenuated. Linear spline models can be generalized in a straightforward manner to accommodate more complex exposure–response relations by using additional knots to generate a multipiece linear spline model. In addition, quadratic and cubic splines may be used instead of linear ones. Quadratic and cubic splines with multiple knots are commonly used to fit exposure–response data in epidemiology (Eisen et al. 2004; Harrell et al. 1988; Steenland and Deddens 2004). These more complex spline models have disadvantages for risk assessment in that their shape is dependent on multiple knot choices, their parameters are not easily interpretable, and they are not linear in the low-dose region. However, such models may help determine an approximate shape for subsequent fitting of a parametric curve that may be more useful for risk assessment. Simpler polynomial or power models without separate pieces (i.e., that are not splines) do not require knot choice (Greenland 1995; Stayner et al. 1997), nor do linear-exponential models that have been used in radiation research (Schneider and Walsh 2008).

Another model of potential interest is the Michaelis-Menten model of enzyme kinetics, in which the rate of reaction tails off as substrate concentration increases (Lehninger 1982). However, as previously noted, exposure–response curves based on these models may be supralinear in the low-dose region when the exposure–response attenuates at high exposures.

Cohort data or nested case–control data, which are typically the basis of environmental or occupational risk assessment using human data, are usually fit using either survival analysis, or conditional logistic regression, where data are grouped in risk sets matched on time via Cox regression. Both linear RR and log-linear RR models for nested case–control data can be fit using common statistical programs such as SAS. Because these models are fit using the same likelihood (Cox’s partial likelihood), their likelihoods can be compared (Langholz and Richardson 2010). Here we provide illustrations of the application of a variety of RR models using data from a cohort study of workers exposed to the sterilant gas ethylene oxide (EtO).

## Methods

Briefly, we previously conducted a cohort analysis, with a focus on breast cancer, of 7,576 women exposed to EtO while sterilizing medical supplies (see Steenland et al. 2003 for details of this study). Here we focus on the 5,138 women from that cohort who we were able to interview; of these women, 232 had been diagnosed with breast cancer. Estimated daily exposures to EtO (average intensity) across different jobs and time periods ranged from 0.05 ppm to 77 ppm—the current U.S. Occupational Safety and Health Administration standard is 1 ppm (U.S. Department of Labor 2007), with an average duration of exposure of 11 years and a median of 7 years (range, 1–50 years). Cumulative exposure was calculated by multiplying duration of exposure by intensity of exposure for each job or time period and summing across different jobs and time periods, with a 15-year lag period such that exposure within 15 years of case diagnosis (or the corresponding date in noncases) was excluded from analyses. The 15-year lag was optimal (best fitting) in log-linear models (Steenland et al. 2003), and we used it again here for linear RR models for consistency.

Models were fit using Cox regression, in which each case was compared with its matched risk set (with matching based on white or nonwhite race), cumulative exposure was time dependent, and the time variable was age. Analyses included the following potential confounders: parity (continuous), date of birth, and family history of breast cancer in first-degree relatives (yes/no). In the linear RR models, one has the choice of combining additive terms for the main exposure with multiplicative terms for covariates, for example, RR = (1 + β_1_X_1_) × exp(β_2_X_2_) × exp(β_3_X_3_), where X_2_ and X_3_ represent covariates, or of using a strictly additive model for both covariates and exposure (e.g., RR = 1 + β_1_X_1_ + β_2_X_2_ + β_3_X_3_). We present results for the strictly additive model, because an inspection of model likelihoods indicated that this type of linear RR model fit the data best. SAS NLP (version 9.1; SAS Institute Inc., Cary, NC) was used for analyses of both log RR models and linear RR models, as suggested by Langholz and Richardson (2010). As noted, the SAS procedure provides comparable model likelihoods for both types of models.

We present a variety of log RR and linear RR models, as described below, including a categorical exposure model (deciles), a linear (untransformed) exposure model (e.g., RR = 1 + cumexp), a model using the log transformation of exposure [(e.g., RR = 1 + log(cumexp)], a model using a square-root transformation (e.g., RR = 1 + square root (cumexp), a two-piece spline model, a linear-exponential model (used only in the linear RR case), and a Michaelis–Menten model. The square-root transformation fits curves that attenuate at high exposures and was chosen to represent the class of polynomial models.

We restricted ourselves to relatively simple statistical models and did not use any biologically based models (e.g., two-stage models), which are not commonly used for risk assessment using human data.

In categorical analyses, exposure was classified into 11 groups, with the reference group including cases and noncases who had no EtO exposure > 15 years before the case was diagnosed with breast cancer (62 breast cancers), and 10 groups with cumulative exposures > 0 with cut points selected such that an approximately equal number of breast cancer cases (*n* = 17) occurred in each decile: 1–355, 356–842, 843–1,361, 1,362–2,187, 2,188–3,772, 3,773–5,522, 5,523–7,891, 7,892–14,483, 14,484–25,112, and > 25,112 ppm-days.

The linear spline model had the form (linear RR version) of RR = 1 + β_1_ × cumexp + β_2z_ × maximum (0, cumexp – knot), where the value corresponding to maximum (0, cumexp – knot) is either the cumulative exposure value less the exposure level used to define the knot, or 0 if the cumulative exposure is less than the value of the knot. The slope of the first exposure–response segment based on the model is represented by β_1_, whereas the slope of the second segment is β_1_ – β_2_. Knots for the two-piece spline model were chosen to maximize the log likelihood after trying knots at 100 ppm-day intervals. In our case, there was a clear optimal knot at 5,800 ppm-days (linear RR model). [See Supplemental Material, Figure 1 (doi:10.1289/ehp.1002521).]

The linear-exponential model had the form RR = 1 + (β_1_ × cumexp) × exp(β_2_× cumexp). This model has been used in radiation studies (Schneider and Walsh 2008) as well as in modeling other human carcinogens (e.g., arsenic) (Lubin et al. 2008) and allows for the linear exposure–response in the usual linear RR model to be modified to exhibit various degrees of curvature.

We also fit the Michaelis–Menten model of enzyme kinetics, in which the rate of reaction tails off as substrate concentration increases (Lehninger 1982). The model has been used rarely in epidemiology, but it is simple to implement, because it merely involves the transformation of exposure and the estimation of one additional parameter to describe the exposure–response relationship. For the linear RR model, the model is RR = 1 + β_1_ × [cumexp/(cumexp + k)], where k represents a constant, analogous to the Km used in biochemistry for enzyme-substrate kinetics, and is estimated from the data.

Another option, not often used in etiologic research but used in studies of the effects of radiation (e.g., Little 2008), is not an RR model at all, but an excess absolute risk (EAR) model, also referred to as an additive Poisson model. This model has the form R_exp_ = R_nonexp_ + β × cumexp. We fit this model following the presentation of Boshuizen and Feskens (2010), who present SAS code for the GENMOD procedure to fit this model via Poisson regression. Poisson regression requires categorized data for rates, unlike the Cox regression models used to fit the linear RR and log RR models here. We cross-categorized events and person-time by age, cumulative exposure (lagged 15 years), and calendar time for these analyses and compared the EAR model with the log RR model. We also used a scale-parameter statistic that adjusted for over- or underdispersion.

Excess risk for environmental exposure to EtO according to each model was calculated using life table techniques accounting for all-cause mortality and applying an adaptation of the method described in the 1988 Biological Effects of Ionizing Radiation (BEIR) report (BEIR 1988). Because the exposure of interest was environmental (i.e., risk to the general public due to EtO in ambient air) and was assumed to begin at birth but with a 15-year lag, risk was effectively considered to begin at age 15 years. More details on the excess risk calculation can be found in Supplemental Material (doi:10.1289/ehp.1002521) and also in Appendix C of the 2006 draft U.S. EPA cancer risk assessment for EtO (U.S. EPA 2006). Briefly, background mortality rates from U.S. vital statistics for 2000 (Centers for Disease Control and Prevention 2010) were stratified by 5-year age groups, and the probability of surviving each age interval was calculated. For incidence calculations, we calculated survival during the interval so that it was the product of the probability of surviving to the interval without a diagnosis of breast cancer and the probability of not getting incident breast cancer during the interval. Surveillance, Epidemiology and End Results Program (SEER; National Cancer Institute 2010) rates for incident breast cancer for 1997–2001 were similarly stratified by 5-year age groups. Then, for each age interval, we calculated the cumulative probability of getting incident breast cancer during the interval, given survival without breast cancer up to that interval. These age-specific probabilities of getting incident breast cancer were then summed across age groups to get the background lifetime (≤ age 85 years) risk of developing breast cancer (result: 0.147). We repeated this same procedure for the exposed group, except that the age-specific background incidence rates of breast cancer were multiplied by the rate ratio predicted by the exposure–response model for the cumulative exposure for that age group. The age-specific cumulative exposures were themselves derived by assuming a constant exposure intensity (set at a given level), which then accumulated daily with a 15-year lag (i.e., starting at age 15 years). Lifetime risk for the exposed population was again the sum of the conditional probabilities of getting breast cancer in each age-group interval, and excess risk was derived by subtracting the risk in the nonexposed population from the risk in the exposed (divided by 1 – lifetime risk in nonexposed). The EC_01_ (i.e., the estimated constant lifetime exposure level associated with a 1% excess lifetime risk of developing breast cancer) is determined by iteratively varying the exposure level until a lifetime excess risk of 0.01 is obtained. Similarly, a 95% one-sided lower confidence limit (LEC_01_) is estimated using the upper one-sided confidence limit of the rate ratio (β + 1.64 × standard error of β) and iterating until the exposure resulting in a 1% excess risk is found.

As noted by Langholz and Richardson (2010), in linear RR models the profile likelihood may be less likely to be approximately normal than in log-linear models, so that *p*-values based on the Wald statistic and the change in log likelihood may differ, and Wald-type confidence bounds may diverge from bounds derived from the profile likelihood. We found such divergence in our linear RR models and used bounds from the profile likelihood to estimate the LEC_01_ for all models except the two-parameter linear-exponential model, where we used the delta method to derive the variance of the RR and the upper one-sided 95% confidence intervals (CIs).

## Results

Table 1 gives the model fit results for the log RR and linear RR models, respectively. Overall, the linear RR models fit the data better than the log RR models, based on the corresponding model likelihoods. Based on the Akaike information criterion (AIC; Akaike 1974), which is a measure of model fit, where a lower value signifies a better fit, the linear RR model using the two-piece linear spline fits better than all other models except the square-root model, which provided the best global fit among both log-linear and linear RR models.

Results for Michaelis-Menten model are not shown. Graphically, this model looked almost identical to the square-root model, but the goodness of fit was not as good as the fit for the square-root model. The AIC was 1951.3; the best estimated k was about 13,000 ppm-days.

The EAR (additive Poisson) model did not fit as well as the RR models. Because the Poisson model requires categorized data and has a different likelihood than do the Cox models used elsewhere here, it is not possible to directly compare the goodness of fit between the Poisson EAR model and the Cox RR models. Therefore, we compared the AIC from an EAR model with the AIC from a log RR Poisson model. The EAR model fit considerably less well than the log RR Poisson model (AIC 1918 vs. 1867; smaller is better). The scale parameter was close to 1 with both models. Consequently, we do not present quantitative risk estimates based on the EAR model.

**Figure 1:**
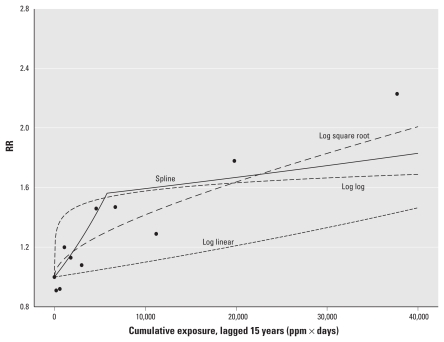
Log RR models for breast cancer incidence and for EtO exposure. Log RR models as used here have the form log RR = β_1_(cumexp). Spline refers to a two-piece spline log RR model with a single knot at 5,800 ppm-days. Log square-root, log-log, and log-linear models refer to log RR models with exposure square root transformed, log transformed, or untransformed, respectively. Individual points indicate estimates from a model with exposure categorized into deciles among the exposed (reference = no exposure, points graphed at the midpoint of each exposure interval).

**Figure 2:**
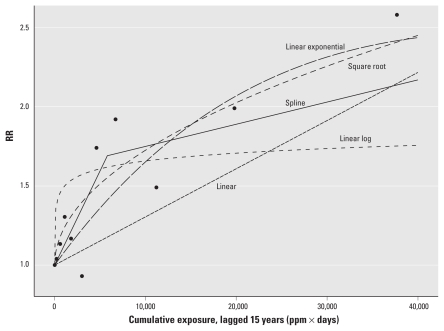
Linear RR models for breast cancer incidence and EtO exposure. Linear RR models as used here have the form RR = 1 + β_1_(cumexp). Spline refers to a two-piece spline linear RR model with a single knot at 5,800 ppm-days. Square root, linear-log, and linear models refer to the linear RR models with exposure square root transformed, log transformed, or untransformed, respectively. The linear-exponential model has the form RR = 1 + {β_1_(cumexp) × exp[β_2_(cumexp)]}. Individual points indicate estimates from a model with exposure categorized into deciles among the exposed (reference = no exposure; points graphed at the midpoint of each exposure interval).

Figures 1 and 2 show the results for the log RR and linear RR models graphically. The shapes of the curves for corresponding log RR and linear RR models are similar. RR estimates based on the categorical models do not follow a monotonic trend with increasing exposure, but the overall pattern suggests increasing RRs with increasing exposure, with some attenuation at the highest exposure decile (i.e., a line fit through the other deciles would pass well above the highest decile). Note that although the cut points for forming decile categories were the same in Figures 1 and 2, the linear RR model gives different categorical results than the log-linear model. Modeling exposure as a simple continuous term appears to underestimate the exposure–response relationship suggested by the categorical point estimates from corresponding log RR and linear RR models. Modeling log-transformed exposure produces log RR and linear RR model estimates that conform reasonably well to the categorical point estimates, but the exposure–response curve is very steep in the low-dose region. Estimates from the two-piece spline models appear to conform well to the categorical results and also suggest a pronounced exposure–response slope for exposures below the knot (< 5,600 ppm-days). The square-root log RR and linear RR models also fit the data well but generate a supralinear shape (although less pronounced than the log-transform model) in the low-dose region.

Table 2 presents the resulting exposure levels that correspond to a 1% lifetime extra risk of breast cancer (EC_01_) based on the five different linear RR models, as well as the LEC_01_ based on using the upper one-sided 95% bound for the exposure–response coefficient. The linear exposure model, with its low slope, had the highest EC_01_, whereas the log-transformed exposure model had the lowest, and the two-piece spline had an EC_01_ between the two. The EC_01_ for the two-piece spline resulted entirely from the first segment of the spline model, because the 1% excess lifetime risk resulted from cumulative exposures < 5,800 ppm-days, that is, within the range of exposures corresponding to the first segment of the estimated exposure–response curve.

### Sensitivity analyses

**Figure 3:**
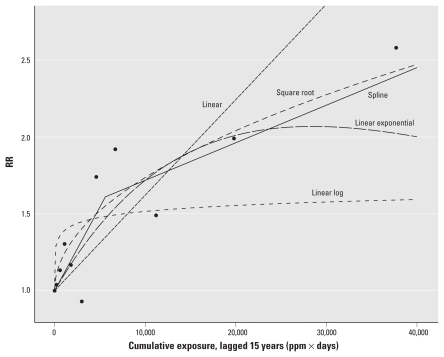
Linear RR models with top 5% of exposure eliminated (> 21,219 ppm-days). Models correspond to those described in Figure 2.

We conducted several analyses to evaluate the sensitivity of results to different model specifications, including the use of a three-piece linear spline model with a second knot at 7,200 ppm-days, which was the optimal location based on a comparison of model fits. However, this model did not significantly improve the fit to the data over that of a two-piece linear spline model (data not shown). We also evaluated truncated linear RR models after excluding observations in the top 5% of the exposure distribution where the most extreme outliers in cumulative exposures occurred. The model of the untransformed (linear) exposure was the most affected by this exclusion, which resulted in a sharp increase in slope indicating its sensitivity to data in the high-exposure range (Figure 3). The EC_01_ for the truncated data changed substantially for the linear model (from 0.040 to 0.020 ppm, a 50% decrease) but less for the log-transform model (from 0.0005 to 0.0006 ppm, a 20% increase) and for the two-piece model (from 0.0010 to 0.0011 ppm, a 10% increase); we observed no change for the linear-exponential model or the square-root model (data not shown).

In addition, we fit the linear RR model after excluding all observations with exposures above 5,800 ppm-days, the location of the knot used in the two-piece spline model, which eliminated the top 20% of exposures. The slope of the exposure–response curve for exposure modeled as an untransformed linear variable was similar (8% higher) to the slope of the lower piece of the two-piece spline model in Figure 2, but its variance was 34% higher (data not shown). This finding illustrates that the slope of the estimated low-dose exposure–response relationship based on the two-piece spline model is not (as might be expected) the same as the slope that would be obtained by simply truncating the data at the preferred knot, because the two segments of the curve produced by the spline model covary, so that one influences the other.

We believe it is generally preferable to use the complete data set to evaluate the low-dose region rather than some truncation of the data that would allow an untransformed linear model to fit well for two main reasons: to avoid some arbitrary point to truncate the data (in the two-piece model, the likelihood function provides an objective mechanism for knot selection), and to avoid information in the upper-dose range that can affect the slope and variance of the exposure–response relationship in the low-dose range.

We also conducted analyses looking at a possible effect of dose rate (using the linear RR model), which for some carcinogens modifies the effect of cumulative dose (e.g., Lubin et al. 2008). Specifically, we divided the observations into high- and low-dose rate groups defined as an average intensity of exposure < 1 ppm and > 1 ppm, which is the current occupational standard and which divided the cases into approximately equal groups. Interaction terms between average intensity (dichotomous indicator term) and either linear (untransformed) exposure or the square root of exposure were not close to statistical significance (*p* = 0.40 and 0.57, respectively). These results indicate that the effect of cumulative dose did not change significantly according to high- or low-dose rate.

## Discussion

Our goal here has been to illustrate key issues in risk assessment related to the choice of a statistical model when risk attenuates at high exposures. We have chosen to rely on relatively simple, easily interpretable models that may be appropriate to risk assessors operating in a public health setting, without entering into a comprehensive discussion of statistical modeling of exposure–response trends. Outside the realm of risk assessment, a wide variety of options are available for choosing the best model, but we argue that these choices are somewhat more limited if they are to conform to the needs of the risk assessor, especially if the goal is estimation of permissible environmental levels rather than occupational levels. Then the shape of the curve in the low-dose region is key. When there is a choice of different models that fit the data well, models that are approximately linear in the low-dose region are preferred, in part because they will not be highly sensitive to the relatively arbitrary choice of a point of departure for low-dose linear extrapolation. The point of departure is particularly sensitive to the shape of the curve in the low-dose region, whereas overall model fit pertains to the validity of the model across the entire exposure range.

Occupational studies for a wide variety of chemicals, often used for risk assessment for both occupational and environmental settings, have frequently shown exposure–response relationships in which the risk of cancer plateaus or attenuates at high exposures (Stayner et al. 2003). A number of reasons have been advanced to explain such attenuation, including depletion of susceptible individuals in the population, saturation of biological processes, misclassification of exposure at high levels, and the healthy worker survivor effect. One might argue that the principal reason for such attenuation is increased misclassification and mismeasurement of exposure at high levels, in which case it might make more sense to attempt to correct the exposure–response data for measurement error than to model mismeasured data. There are several arguments against this approach. First, from a practical standpoint, it is rare that a gold standard of well-measured data is available to estimate the direction and amount of mismeasurement. Second, there is no reason *a priori* to assume that measurement error is the principal reason for observed attenuation, and it is not necessarily clear that attenuation will result from this error. Measurement error that is nondifferential and of the Berkson type (Armstrong 1998; as in occupational studies where the mean job-specific exposure is assigned to all workers in that job) with constant error variance across exposure levels has been shown in RR models to result in a downward bias on the exposure–response relationships in RR models (Armstrong 1990). However, others have shown in log-linear RR models that increased mismeasurement of high exposures versus low exposures does not necessarily lead to estimates of exposure–response relationships biased to the null and can, in fact, lead to overestimates of exposure–response relationships (Steenland and Deddens 2000). Whether one might expect attenuation due to mismeasurement in linear RR models under this scenario is not clear.

We argue that a two-piece spline model is a good candidate for risk assessment when there is risk attenuation at high exposure levels. We have used data on breast cancer incidence and EtO exposure, which exhibit attenuation, to illustrate the two-piece spline model; however, as noted above, there are many other examples where this approach would be suitable. We also argue that additive models (linear RR) offer some advantages for risk assessors, namely, easy interpretability and a strictly linear form in the low-dose region of interest.

When there is attenuation, the risk assessor has a number of choices. For example, if the exposure–response curve plateaus as exposure increases, the risk assessor may face a dilemma of whether to consider all the data or make some relatively arbitrary decision to exclude high-exposure data altogether from the exposure–response model to focus on the low-exposure region that is the most informative and relevant for estimating low-dose risk (e.g., from environmental exposures). We have argued that the two-piece linear model provides one solution to this dilemma that avoids the need to make a subjective decision about what to define as high exposure and has the added benefit of a linear exposure–response curve in the low-exposure region.

One potential disadvantage to the two-piece spline model can arise in choosing the knot based on the likelihood, particularly when data are sparse. Sometimes the best likelihood for choosing the knot is not much better than another, that is, the profile likelihood is fairly flat or has several local maxima. This will also occur when the data are not sparse but the two-piece spline model does not provide a good fit to the data.

The variety of models illustrated here provide a statistically reasonable fit to the overall breast cancer morbidity data considered, but they have very different shapes in the low-exposure region, resulting in very different EC_01_ estimates. The two-piece spline model yields estimates between the more extreme results obtained from the log-transformed model (very supralinear in the low-exposure region) and the untransformed (linear in exposure) model (too sublinear in the low-exposure region). The square-root model also is somewhat supralinear in the low-dose region, resulting in a low EC_01_.

An alternative to choosing the best of a series of models that fit the data reasonably well is model averaging, in which the exposure–response results from different models are averaged using some sort of weighting scheme. When these models have the same parametric form, a weighted average of the parameters can be calculated using weights determined from the likelihood in a frequentist setting or from Bayesian posterior probabilities for the parameters (assumed to be random variables). Schwartz et al. (2008) provide an example from air pollution where a five-knot linear spline model was used with different lags on pollution exposure. A series of models was run in which the change in slope at each knot was constrained in different ways (e.g., a threshold model in which the slope of the first piece of the linear model was constrained to be 0). The parameters for slope in each piece were then averaged using as weights the Bayesian posterior probabilities, with noninformative priors or priors favoring a nonthreshold model over a threshold model. Wang et al. (2004) provide a good discussion of this same type of model averaging in a slightly different context. They show via simulation how Bayesian model averaging can outperform backward and forward selection techniques to pick the best model from a large set of candidate predictors, then show how parameter estimates for a given set of predictors from the best models can be averaged using posterior probabilities to allow inference about these parameters while reflecting the uncertainty of model selection.

When the models have different parametric forms, as in our case (e.g., square root–transformed model, two-piece model), model averaging might be done via calculation of different EC_01_ values and taking a weighted average across them using model likelihoods or Bayesian posterior probabilities. Noble et al. (2009) have used model averaging to calculate a benchmark concentration, analogous to an EC, in a study of lung function and coal dust, using as weights Bayesian posterior probabilities with noninformative priors, across linear, square root, and quadratic models, with the selection of a set of covariates, such as height and weight, from among six possible ones. The subset of best models used in the model averaging, out of all possible models, was chosen using a type of selection rule called Occam’s window (Noble et al. 2009), based on the posterior likelihood for the model. One difficulty which arises is deciding what the necessarily arbitrary cut point will be for excluding some range of poorly fitting models from the average.

Model averaging makes more sense, in general, in situations where the results of different models are similar, for example, using a set of best models chosen via Occam’s window. Assuming a relatively broad inclusion rule, a model result that is an outlier on one side of the central tendency may have undue influence on the model average if the fit of the outlier model is reasonable (thus increasing the weight of its contribution to the average across all models) and if it is not balanced by an outlier on the other side of the central tendency. In our data, we found that different models, all of which fit the data reasonably well, produced very different estimates of EC_01_. Although an AIC-weighted average of EC_01_ estimates from Table 2 yielded a model-average EC_01_ of 0.013, which was not far from the estimates based on the two-piece linear and linear-exponential models, this occurred because the two outliers (from the linear and log-transformed exposure models) were on opposite sides of 0.013—a situation that might not have occurred if we had overlooked one model form or another. Furthermore, models that are not linear in the low-dose region (which is approximately 0–1,000 ppm in our data) will lead to very different estimates of permissible exposure levels, depending on the somewhat arbitrary choice of EC_01_ or EC_05_ or EC_001_, for example, as the point of departure for low-dose extrapolation. In our case, limiting ourselves to models that were approximately linear in the low-dose region implies limiting ourselves to the linear exposure model, the two-piece linear model, and perhaps the linear-exponential model. In our view the linear model is an outlier that did not fit the data as well as the other two models, and we would not advise averaging these three models. In sum, from both a public health and risk assessment perspective, we believe it best to choose a best model based on a combination of model fit and a desire that the resulting model be linear or close to linear in the low-dose region.

## Figures and Tables

**Table 1:** Log RR and linear models.

Exposure	df	*p*-Value^a^	−2LL	AIC^b^
Log RR models				
Log transformed	1	0.03	1944.17	1956.17
Untransformed (linear)	1	0.04	1944.68	1956.68
Categorical	10	0.29	1936.91	1966.91
Two-piece linear spline	2	0.01	1940.48	1954.48
Square root transformed	1	0.005	1941.03	1953.03

Linear RR models				
Log transformed	1	0.0030	1942.27	1954.27
Untransformed (linear)	1	0.0096	1940.26	1952.26
Categorical	10	0.1249	1933.94	1963.94
Two-piece linear spline	2	0.0023	1936.94	1950.94
Square root transformed	1	0.0007	1937.49	1949.49
Linear exponential	2	0.0035	1937.78	1951.78

Abbreviations: df, degrees of freedom; −2LL, −2 log likelihood.

a *p*-Value for chi square, the change in −2LL by the addition of exposure variable(s) to model.

b All models included three indicator variables for date of birth, one for family history, and one for parity. The −2LL for a model that included only the covariates date of birth, parity, and first-degree relative with breast cancer was 1948.93, and the −2LL for a model with no covariates was 1967.81. The AIC is derived as −2LL + 2 × (number of parameters estimated in model); a smaller AIC value indicates a better fit, adjusted for different number of parameters in the model.

**Table 2:** EC_01_ estimates for linear RR models.

Exposure	EC_01_ (ppm)	LEC_01_^a^ (ppm)
Untransformed (linear)	0.0400	0.0165
Log transformed	0.00005	0.00002
Two-piece linear spline	0.0100	0.0039
Square root transformed	0.0016	0.0003
Linear exponential	0.0113	0.0058

a Lower CI for EC_01_, using upper one-sided 95% confidence limit for exposure–response coefficient, as determined using profile likelihood for single parameter models and for the first piece of the two-piece spline model, applicable in the low-dose region of interest (below the knot, where the second parameter is 0). For the two-parameter linear-exponential model, we used the delta method to derive the variance of the RR and upper one-sided 95% CI using Wald-type variances for each parameter, given the added computational complexity of deriving profile-based bounds using the joint likelihood for both parameters.
